# Supplementary material for: Effects of physical activity and exercise on well-being in the context of the Covid-19 pandemic
Source: PLoS One. 2022 Jan 26;17(1):e0260465. doi: 10.1371/journal.pone.0260465 (PMC8791524; doi:10.1371/journal.pone.0260465)
Supplement: Attachment — Submitted filename: CartaAosRevisores.docx [file pone.0260465.s002.docx]

Response to the Reviewers,

First, we want to thank you for all considerations about our article. We think there were very important to make this work better to publication. Because of that we considered all the reviewers’ suggestions.

This is a revised version of the manuscript that addresses all the points raised during the review process. There are include this items:

1. This letter that we respond to each point raised by the academic editor and reviewer(s).
2. A marked-up copy of our manuscript that highlights changes made to the original version.
3. An unmarked version of your revised paper without tracked changes.

1. Please ensure that your manuscript meets PLOS ONE's style requirements, including those for file naming. The PLOS ONE style templates can be found at

WE PUT THE MANUSCRIPT IN THE TEMPLATES AT THIS ADRESS - <https://journals.plos.org/plosone/s/file?id=ba62/PLOSOne_formatting_sample_title_authors_affiliations.pdf>

2. Thank you for stating the following in the Acknowledgments and the Founding Source Sections of your manuscript:

THE INFORMATION ABOUT FUNDING WAS IN THE Funding Statement section of the online submission form.

"The funders had no role in study design, data collection and analysis, decision to publish, or preparation of the manuscript."

Please include your amended statements within your cover letter; we will change the online submission form on your behalf.

[Note: HTML markup is below. Please do not edit.]

Reviewers' comments:

**Comments the reviewer's**:

1. Is the manuscript technically sound, and do the data support the conclusions?

The manuscript must describe a technically sound piece of scientific research with data that supports the conclusions. Experiments must have been conducted rigorously, with appropriate controls, replication, and sample sizes. The conclusions must be drawn appropriately based on the data presented.

Reviewer #1: Yes

Reviewer #2: Yes

Reviewer #3: Partly

*AUTOR: because the reviewer #3, we reorganized the conclusions to draw appropriately.*

2. Has the statistical analysis been performed appropriately and rigorously?

Reviewer #1: Yes

Reviewer #2: Yes

Reviewer #3: I Don't Know

*AUTOR: in this item, the major of the reviwers considerated the statistical analysis well, so that was maintened in the final version*

3. Have the authors made all data underlying the findings in their manuscript fully available?

The [PLOS Data policy](http://www.plosone.org/static/policies.action#sharing) requires authors to make all data underlying the findings described in their manuscript fully available without restriction, with rare exception (please refer to the Data Availability Statement in the manuscript PDF file). The data should be provided as part of the manuscript or its supporting information, or deposited to a public repository. For example, in addition to summary statistics, the data points behind means, medians and variance measures should be available. If there are restrictions on publicly sharing data—e.g. participant privacy or use of data from a third party—those must be specified.

Reviewer #1: No

Reviewer #2: Yes

Reviewer #3: Yes

4. Is the manuscript presented in an intelligible fashion and written in standard English?

PLOS ONE does not copyedit accepted manuscripts, so the language in submitted articles must be clear, correct, and unambiguous. Any typographical or grammatical errors should be corrected at revision, so please note any specific errors here.

Reviewer #1: Yes

Reviewer #2: Yes

Reviewer #3: Yes

*AUTOR: thats ok in this item.*

5. Review Comments to the Author

Please use the space provided to explain your answers to the questions above. You may also include additional comments for the author, including concerns about dual publication, research ethics, or publication ethics. (Please upload your review as an attachment if it exceeds 20,000 characters)

Reviewer #1: The manuscript covers a very interesting topic and the approach used by the authors is quite robust. However, the way it is written and structured makes it hard for the reader to follow. In addition, some information needs to be better explained by the authors. Detailed comments:
- The manuscript would be both more compelling and useful to a broad readership if the authors moved beyond providing a descriptive narrative of the data analyzed in an assembled way and be more forthright and clear in presenting the instruments and the results (maybe using tables).
- The number of acronyms should be considerably reduced, as it makes the text hard to follow, particularly in the methods section. By the way, the acronym of Positive Affect should be PA and not AP.
- It is not clear if all participants answered all the questionnaires’ items.
- The way results are presented is rather confusing: subtopics should be added to direct the reader.
- The correlation between the sociodemographic data collected and the instruments should be clearer and better explored.
- A "Limitations" section at the end of the "Discussion" and as well as a “Conclusion” section should be included. In the “Conclusion” section, policy implications of the results achieved should be added: conclusion section was inclued.

**Response reviewer #1:** We make changes in the way it is written, the structured is change to make the reading lighter. In the data analyzed we change the way to presente the instruments and the results to make more clear. The number of acronyms was reduced and the error has been corrected in the acronym of Positive Affect that should be PA and not AP. We make clear tha the all participants answered all the questionnaires’ items. To attend the revisor we added subtopics in the results. In the results we make better the correlation between the sociodemographic data collected and the instruments. We included the policy implications of the results. All this change can be seen in the archive marked-up copy of our manuscript that highlights.

Reviewer #2: The manuscript is of general interest and conducted with a rigorous research approach. I am satisfied with the work done by the authors. I have only the following minor comments to rise.

**Response reviewer #2: in red**

ABSTRACT
- The authors reported the acronym SWB without having defined it first: Has been solved
- Please provide age of the sample and gender distribution: it was inclued
- Why did the authors only report strength training results? Authors should better report the main findings found: all the results was inclued in the abstract

INTRODUCTION
- The introduction is too long and dispersed. Authors should streamline it and make it converge on the topics related to the study (for example, the description of the symptoms of COVID-19 or the difference between symptomatic and asymptomatic is not necessary for the purpose of the study): the introduction was reduce and more direct.
- Some sentences are not supported by bibliographic sources (for example the following sentence: “However, the same physical distance, which protects COVID-19 from spreading, may have an impact on mental health and the well-being of the population.” For the latter statement I suggest considering the following references:
1) Giustino, V. et al. Physical Activity Levels and Related Energy Expenditure during COVID-19 Quarantine among the Sicilian Active Population: A Cross-Sectional Online Survey Study. Sustainability 2020, 12, 4356.
2) López-Bueno, R. et al. Health-Related Behaviors Among School-Aged Children and Adolescents During the Spanish Covid-19 Confinement. Frontiers in paediatrics 2020, 8, 573.
We appreciate the references and put then in the manuscript

DISCUSSION
- In the discussion, same sentences concerning the PAWEC should be reported in the methods section (for example the following sentence: “the scale as a whole showed Cronbach’s alpha above 0.70, which was considered enough to assume good internal consistency. The instrument, then, proved to be reliable and well structured”): That information about PAWEC is in the discussion because this was the first time that scale was applied, so these information was results about the psicometric informations. Is important to mention that Only after the application we knowed about the tree dimensions of PAWEC: exercise effects (positive or negative affect of exercise for people in quarantine), cognition (cognitive variables which entail understanding reasons and importance of exercise during confinement) and mood (motivational and emotional aspects involved in exercising during social isolation).

- Before describing the results found, the authors should report what their hypothesis was: we apreciate this sugestion, so we puted our hypothesis before the results found

- The authors failed to report strength and limitations of the study. Among the latter, the self-reported based instrument should be mentioned: in the end of the article we mentioned this

Reviewer #3: 1. The date was collected for March 31st and April 2nd, 2020. Thats perhaps a very small time for a study of this magnitude to be conducted: we justify this short time of data collection, because we wanted to identify the feelings at the beginning of the pandemic

2. the word SWB ?? is present in the abstract, what does it stand for ?: we put the complet name: Subjective Well-Being.

3. The sentence "However, it is unclear whether people in quarantine are able to engage in such frequency and regularity of exercise and if this practice is enough to provide SWB." Was it also measured/investigated in the present research: yes, because we finded that the frequency and regularity before the isolation is importante to provide SWB in the isolation.

4. From the methodology I feel three Instruments were administered to the same population, Do the authors think RANOVA/MANOVA should had been used to control type 2 errors?
5. Another question that arises is how the author controlled the Intensity or administered the intensity for exercises for the participants during the pandemic , as specific intensity will produce specific results: in fact we controlled the frequency and regularity of exercise, not the intensity
6. Refer Can moderate intensity aerobic exercise be an effective and valuable therapy in preventing and controlling the pandemic of COVID-19?Med Hypotheses. 2020 Oct; 143: 109854. and Physical exercise as a tool to help the immune system against COVID-19: an integrative review of the current literature, Clinical and Experimental Medicine (2020). Should be included and discussed in the article: we appreciated and this was inclued
7. Limitations if any: we put the limitations
8. I think there is a need to revise the need or gap for the study which needs to be more emphatic so does the conclusion.

6. PLOS authors have the option to publish the peer review history of their article ([what does this mean?](https://journals.plos.org/plosone/s/editorial-and-peer-review-process#loc-peer-review-history)). If published, this will include your full peer review and any attached files.


If you choose “no”, your identity will remain anonymous but your review may still be made public.


**Do you want your identity to be public for this peer review?** For information about this choice, including consent withdrawal, please see our [Privacy Policy](https://www.plos.org/privacy-policy).

Reviewer #1: No

Reviewer #2: No

Reviewer #3: **Yes:** DR SNEHIL DIXIT

[NOTE: If reviewer comments were submitted as an attachment file, they will be attached to this email and accessible via the submission site. Please log into your account, locate the manuscript record, and check for the action link "View Attachments". If this link does not appear, there are no attachment files.]

While revising your submission, please upload your figure files to the Preflight Analysis and Conversion Engine (PACE) digital diagnostic tool, <https://pacev2.apexcovantage.com/>. PACE helps ensure that figures meet PLOS requirements. To use PACE, you must first register as a user. Registration is free. Then, login and navigate to the UPLOAD tab, where you will find detailed instructions on how to use the tool. If you encounter any issues or have any questions when using PACE, please email PLOS at [figures@plos.org](mailto:figures@plos.org). Please note that Supporting Information files do not need this step.

*In compliance with data protection regulations, you may request that we remove your personal registration details at any time.* [*(Remove my information/details)*](https://www.editorialmanager.com/pone/login.asp?a=r)*. Please contact the publication office if you have any questions.*
