# Supplementary material for: Effects of physical activity and exercise on well-being in the context of the Covid-19 pandemic
Source: PLoS One. 2022 Jan 26;17(1):e0260465. doi: 10.1371/journal.pone.0260465 (PMC8791524; doi:10.1371/journal.pone.0260465)
Supplement: Attachment — Submitted filename: Letter-to-Editor.docx [file pone.0260465.s003.docx]

Dear Editor,

In behalf of the other authors, I can say that we appreciate the effort of both Editor and Reviewers in making this manuscript better and ready for publication at Plos One. We tried to tap into the last requirements that were asked. We made a full language and format review with a native English speaker in order to make it suitable for publication. We thank the opportunity and look forward to hearing from Plos One. Below, the list of modifications.

1) Citations were completely reviewed and reorganized according to Plos One format (Vancouver).

2) Language was reviewed by a native English speaker.

3) To improve clarification, the term *disposed* was replaced by *energetic* in Table 1 (pg. 14)

4) Last paragraph of results (pg. 16) was completely rewritten for better understanding of the reader.

5) Last phrase of the first paragraph of Discussion (pp. 16-17) was rewritten due to language issues.

6) First paragraph of the first subsection of the Discussion (pg. 18) was changed to improve understanding.

7) In-text citations were reviewed to be adequate to Vancouver format.
